# Projected COVID-19 Mortality Reduction From Paxlovid Rollout

**DOI:** 10.1001/jamahealthforum.2023.0046

**Published:** 2023-03-17

**Authors:** Mihir Khunte, Soryan Kumar, Joshua A. Salomon, Alyssa Bilinski

**Affiliations:** 1 Warren Alpert Medical School, Brown University, Providence, Rhode Island; 2 Center for Health Policy and Center for Primary Care and Outcomes Research, Stanford University School of Medicine, Stanford, California; 3 Department of Health Services, Policy, and Practice and Department of Biostatistics, Brown School of Public Health, Providence, Rhode Island

## Abstract

This decision analytical model study assesses projections of simulated effects of Paxlovid rollout on hospitalizations and mortality using 10 models.

## Introduction

COVID-19 was the third leading cause of death in the US in 2022, and following FDA approval of Paxlovid (nirmatrelvir-ritonavir), the test-to-treat initiative became a cornerstone of the US pandemic response.^1^ Paxlovid treatment requires testing and initiating medication within 5 days of symptom onset. However, the population-level impact of Paxlovid rollout has not been estimated.^2^ The purpose of this study is to project simulated effects of Paxlovid rollout on hospitalizations and mortality and to quantify the number of COVID-19 tests and Paxlovid courses required for different levels of mortality reduction during a surge comparable to the 2022 winter Omicron wave (WOW) (December 15, 2021, to March 15, 2022).

## Methods

We modeled COVID-19 hospitalization and mortality reductions associated with Paxlovid rollout as the product of (1) the proportion of eligible symptomatic patients tested within 5 days of symptom onset, (2) probability of receiving Paxlovid given eligibility, and (3) Paxlovid effectiveness against hospitalization and mortality risks. Details on quantification of number of required symptomatic tests and required Paxlovid courses are available in eMethods in Supplement 1. For sensitivity analyses, we constructed 10 models (Table) to estimate optimistic and pessimistic bounds for hospitalization and mortality reduction (models 2-3), subpopulation analyses (models 4-6), projections given increased Paxlovid uptake (models 7-8), and tests and Paxlovid courses required (models 9-10). Detailed descriptions for each model are provided in the eMethods and eTables 1-3 in Supplement 1. Institutional review board approval was not required as human participants were not involved per the Common Rule (45 CFR §46). The study followed the CHEERS reporting guideline.

**Table.** Model Parameters and Results

Parameter	Sensitivity analyses on reductions in mortality/hospitalizations								Sensitivity analyses on tests/Paxlovid courses required, eligibility	
	Model 1 base case^a^	Model 2 lower bound^b^	Model 3 upper bound^c^	Model 4 NH uptake^d^	Model 5 vaccinated^e^	Model 6 unvaccinated^f^	Model 7 optimistic uptake = 40%^g^	Model 8 optimistic uptake = 80%^h^	Model 9 low^i^	Model 10 high^j^
Probability patient is tested within 5 d of symptoms	0.78	0.55^k^	0.85^k^	0.78	0.78	0.78	0.78	0.78	0.55^k^	0.85^k^
Probability of CLI	0.03	0.03	0.03	0.03	0.03	0.05^k^	0.03	0.03	0.03	0.05^k^
% Of patients who qualify for Paxlovid	0.38	0.38	0.38	0.38	0.38	0.38	0.38	0.38	0.3^k^	0.49^k^
Duration of symptom episode, d	7	7	7	7	7	8	7	7	8^k^	6^k^
No. of COVID tests per symptomatic individual, mean^l^	1.95	1.95	1.95	1.95	1.95	1.95	1.95	1.95	1.53^k^	2.39^k^
Reduction										
In overall hospitalization due to Paxlovid	0.67	0.67	0.88^k^	0.67	0.7^k^	0.88^k^	0.67	0.67	0.67	0.67
In overall mortality due to Paxlovid	0.81	0.81	0.88^k^	0.81	0^k^	0.88^k^	0.81	0.81	0.81	0.81
Proportion of Paxlovid uptake	0.05	0.01^k^	0.10^k^	0.15^k^	0.05	0.05	0.40^k^	0.80^k^	0.01^k^	0.10^k^
Results										
No. of symptomatic tests during WOW, million	4.8	1.3	9.4	13.9	4.8	7.0	37.6	75.3	0.7	29.5
No. of Paxlovid courses during WOW, million	2.5	0.5	5.4	7.4	2.5	2.5	19.9	39.8	0.4	7.1
Reduction, %										
In hospitalization via Paxlovid rollout	2.7	0.5	7.5	7.7	2.8	3.5	20.9	41.8	0.5	5.7
In mortality via Paxlovid rollout	3.2	0.6	7.5	9.3	0.0	3.5	25.3	50.5	0.6	6.9

Abbreviations: CLI, COVID-like illness; NH, nursing homes; WOW, winter Omicron wave.

^a^ Model 1 shows the base case scenario with expected values for each parameter.

^b^ Model 2 provides a lower bound estimate for reductions in hospitalization and mortality by lowering the probability of testing within 5 days of symptoms and the proportion of the high-risk population taking Paxlovid.

^c^ Model 3 provides an upper bound for reductions in hospitalization and mortality by raising (1) the probability of testing within 5 days of symptoms, (2) the proportion of the high-risk population taking Paxlovid, and (3) the effectiveness of Paxlovid. Note that we obtained 2 possible values for the effectiveness of Paxlovid in the general population. The higher values are used in model 3.

^d^ Model 4 provides an optimistic estimate of Paxlovid uptake by raising the general population level of Paxlovid uptake to that of NHs and provides results at the US population level.

^e^ Model 5 provides an estimate for reductions in hospitalization and mortality for a population that is entirely vaccinated by altering the Paxlovid efficacy in hospitalization and mortality based on those seen in vaccinated and provides results at the US population level.

^f^ Model 6 provides an estimate for reductions in hospitalization and mortality for a population that is unvaccinated by increasing the prevalence of COVID-like symptoms and duration of each symptomatic episode, in addition to altering Paxlovid efficacy in hospitalization and mortality based on those seen in unvaccinated patients. Results are provided at the US population level.

^g^ Model 7 provides an optimistic estimate for reductions in hospitalization and mortality if the US can achieve a Paxlovid uptake of 40%.

^h^ Model 8 provides an optimistic estimate for reductions in hospitalization and mortality if the US is able to achieve a Paxlovid uptake of 80%.

^i^ Model 9 provides a lower bound for the required number of symptomatic tests and Paxlovid courses by raising the (1) duration of each symptomatic episode, and lowering the (2) proportion of patients testing within 5 days, (3) percent of patients qualifying for Paxlovid, (4) mean number of tests per individual, and (5) proportion of the high-risk population taking Paxlovid.

^j^ Model 10 provides an upper bound for the required number of symptomatic tests and Paxlovid courses by lowering the: (1) duration of each symptomatic episode, and raising the (2) proportion of patients testing within 5 days, (3) percent of patients qualifying for Paxlovid, (4) mean number of tests per individual, (5) proportion of the high-risk population taking Paxlovid, and (6) prevalence of COVID symptoms. Note that we arrived at 2 possible values for the prevalence of COVID symptoms. The higher value is used in model 10.

^k^ Values altered from baseline.

^l^ Calculation of the mean number of COVID tests per symptomatic individual did not use data of which SDs can be taken. The mean number of COVID tests per symptomatic individual was calculated algebraically using values found in the literature; a weighted average of the number of symptomatic tests was taken for infected (1.5) and uninfected individuals (2-2.5). For infected individuals, the inverse of the overall sensitivity of the rapid antigen test (0.65) was taken to arrive at 1.5. An additional test was added for uninfected individuals to rule out infection per Centers for Disease Control and Prevention guidelines. The variation in parameters across models resulted from differences in the estimated number of COVID tests for uninfected individuals.

## Results

We estimated that 78% of US cases that will require hospitalization are detected within 5 days of symptom onset, and that uptake of Paxlovid is 5% among eligible infected individuals. Given Paxlovid effectiveness of 67% against hospitalization and 81% against mortality, this corresponds to relative percentage reductions of COVID-19 hospitalization by 2.7% and mortality by 3.2% (Figure).

**Figure.**
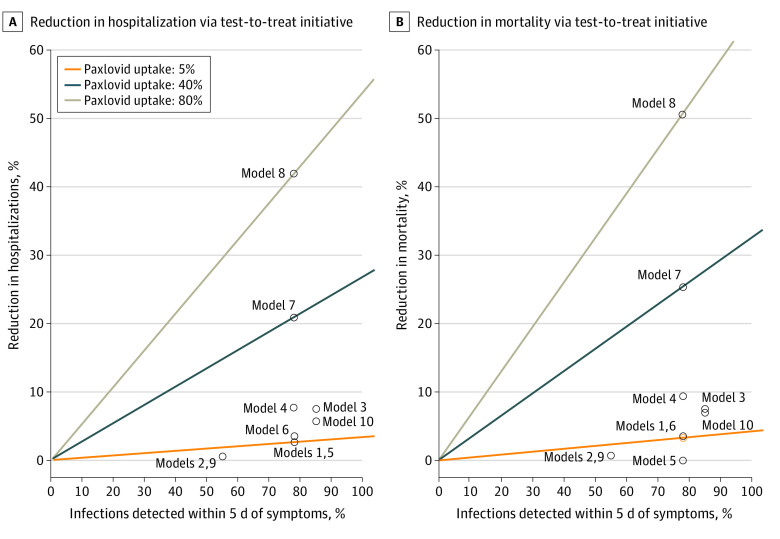
Projected Reductions in Hospitalizations and Mortality Descriptions for models 1 through 10 are given in the footnotes to the Table.

In sensitivity analyses (Table, models 2-3), COVID-19 hospitalization reductions varied between 0.5% and 7.5% and mortality reductions between 0.6% and 7.5%. However, in nursing homes, with higher uptake, we estimate hospitalization and mortality reductions at 7.7% and 9.3% (model 4). If Paxlovid uptake among eligible populations increases to 40% (model 7), we project a 21% reduction in hospitalization and 25% reduction in mortality. If Paxlovid uptake increases to 80% (model 8), we project a 42% reduction in hospitalization and 51% reduction in mortality.

At 5% Paxlovid uptake (model 1), the required number of symptomatic tests and Paxlovid courses needed during the WOW would have been 4.8 million and 2.5 million, respectively, averting 2.7% of hospitalizations and 3.2% of deaths. At 80% Paxlovid uptake (model 8), the required number of symptomatic tests and Paxlovid courses would have been 75.3 million and 39.8 million, respectively, averting 41.8% of hospitalizations and 50.5% of deaths.

## Discussion

In this study, we estimated that had current Paxlovid uptake been achieved in January 2022, 4.8 thousand deaths would have been averted during the WOW. Our estimates suggest that 0.7 to 75.3 million symptomatic tests and 0.4 to 39.8 million courses of Paxlovid are needed for a future Omicron-like wave.

There are limitations to this work. Our parameterization is limited by a dearth of data on Paxlovid uptake. Also, relevant parameters are likely to continue shifting over time due to reduced prescribing restrictions or potential resistance.^3,4^ Nevertheless, in this rapidly changing landscape, we provide a simple, flexible framework for understanding the resource requirements and benefits associated with future expansions of the test-to-treat initiative.

## Notes

Supplement 1. **eMethods.** Supplementary Methods **eTable 1.** Model Parameter Values **eTable 2.** Model Number and Logic for Model **eTable 3.** Formulae for Calculated Parameters in eTable 1

Supplement 2.
Data Sharing Statement

## References

[1] US Department of Health and Human Services. Fact sheet: Biden administration launches nationwide test-to-treat initiative ensuring rapid “on the spot” access to lifesaving COVID treatments. March 8, 2022. Accessed January 27, 2023. https://www.hhs.gov/about/news/2022/03/08/fact-sheet-biden-administration-launches-nationwide-test-treat-initiative-ensuring-rapid-on-spot-access-lifesaving-covid-treatments.html

[2] Centers for Disease Control and Prevention. COVID-19 treatments and medications. 2022. Accessed January 27, 2023. https://www.cdc.gov/coronavirus/2019-ncov/your-health/treatments-for-severe-illness.html

[3] Tanne JH. Covid-19: FDA authorises pharmacists to prescribe Paxlovid. BMJ. 2022;378:o1695. doi:10.1136/bmj.o1695 35803606

[4] Mótyán JA, Mahdi M, Hoffka G, Tőzsér J. Potential resistance of SARS-CoV-2 main protease (Mpro) against protease inhibitors: lessons learned from HIV-1 protease. Int J Mol Sci. 2022;23(7):3507. doi:10.3390/ijms23073507 35408866 PMC8998604

