# Supplementary material for: Projected COVID-19 Mortality Reduction From Paxlovid Rollout
Source: JAMA Health Forum. 2023 Mar 17;4(3):e230046. doi: 10.1001/jamahealthforum.2023.0046 (PMC10024200; doi:10.1001/jamahealthforum.2023.0046)
Supplement: Supplement 1. — eMethods. Supplementary Methods eTable 1. Model Parameter Values eTable 2. Model Number and Logic for Model eTable 3. Formulae for Calculated Parameters in eTable 1 [file jamahealthforum-e230046-s001.pdf]

Supplemental Online Content

Khunte M, Kumar S, Salomon JA, Bilinski A. Projected COVID-19 mortality reduction from Paxlovid rollout. *JAMA Health Forum.* 2023;4(3):e230046.
doi:10.1001/jamahealthforum.2023.0046

eMethods. Supplementary Methods

eTable 1. Model Parameter Values

eTable 2. Model Number and Logic for Model

eTable 3. Formulae for Calculated Parameters in eTable 1

eReferences

This supplemental material has been provided by the authors to give readers additional information about their work.

© 2023 Khunte M et al. *JAMA Health Forum*.

eMethods. Supplementary Methods

We developed a branching model for COVID-19 hospitalization and mortality to determine relative percentage reductions associated with Paxlovid roll-out for a highly transmissible and moderately severe COVID-19 variant such as Omicron. The model was parameterized based on national data and clinical studies. To compute hospitalization and mortality reductions, we assumed all hospitalizations and deaths occur among Paxlovid-eligible individuals. The model accounts for partial population immunity from immunization and natural infection from existing and past variants. The parameters most affected by immune protection from natural infection and vaccination include probability of mortality, hospitalization, and COVID-like symptoms. These parameters were varied extensively to provide lower and higher bounds for how changes in immune protection in the population may impact Paxlovid effectiveness. All analyses were completed using Microsoft Excel 365 and R, Version 3.6.2.

To quantify the number of required symptomatic tests during the 2022 winter Omicron wave (WOW) (12/15/2021-3/15/2022), we multiplied (1) the number of individuals with COVID-like illness (CLI), (2) percentage of Paxlovid-eligible infections, (3) mean number of tests administered per CLI episode, and (4) probability of receiving Paxlovid given eligibility. For the number of required Paxlovid courses during the WOW, we multiplied (1) the number of infections, (2) percentage of Paxlovid-eligible infections, (3) proportion of symptomatic patients tested within 5 days, and (4) probability of receiving Paxlovid given eligibility.

eTable 1: Model parameter values.

Parameter	Base Case Value	Source
Probability patient is tested within 5 days of symptoms	78%	Calculated from COVID-19 Case Surveillance Public Use Data[1]
Probability of COVID-like symptoms in US population	3%	Carnegie Mellon University Delphi Group COVID-Like Symptoms[2]
Percent of patients who are high risk and qualify for Paxlovid	37.6%	Koma et al. Kaiser Family Foundation[3]
US population	333 million	U.S. Population Clock[4]
Duration of symptom episode in days	7	Menni et al. The Lancet[5]
Average of number of tests for symptomatic individuals (infected)	1.5	Calculated from Jegerlehner et al. International Journal of Infectious Diseases[6]
Average of number of tests for symptomatic individuals (non-infected)	2	Jegerlehner et al. International Journal of Infectious Diseases[6] COVID-19 Testing Information from the Centers for Disease Control and Prevention[7]
Average of number of tests for symptomatic individuals (total)	1.95	Calculated from above two rows and 'Proportion of symptomatic patients that are positive for COVID'
Reductions in overall hospitalization due to Paxlovid	67%	Arbel et al. Research Square Preprint[8]
Reductions in overall mortality due to Paxlovid	81%	Arbel et al. Research Square Preprint[8]
Reductions in overall hospitalization due to Paxlovid in vaccinated population	70%	Pfizer Press Release – EPIC Standard Risk Group[9]
Reductions in overall hospitalization due to Paxlovid in unvaccinated population	88%	Hammond et al. New England Journal of Medicine[10]

© 2023 Khunte M et al. *JAMA Health Forum*.

Reductions in overall deaths due to Paxlovid in vaccinated population	0	Pfizer Press Release – EPIC Standard Risk Group[9]
Reductions in overall deaths due to Paxlovid in unvaccinated population	88%	Hammond et al. New England Journal of Medicine[10]
Estimated number of daily infections during omicron wave	1.9 million	Calculated from Institute for Health Metrics and Evaluation COVID-19 Estimated Infections Data[11]
Estimated number of total Infections During omicron wave	169 million	Calculated from Institute for Health Metrics and Evaluation COVID-19 Estimated Infections Data[11]
Proportion of positive patients that are symptomatic	59.5%	Calculated from Ma et al. JAMA Infectious Diseases[12]
Proportion of symptomatic patients that are positive for COVID	11%	Calculated from Ma et al. JAMA Infectious Diseases[12]
Total courses of Paxlovid utilized (7/09/22 – 7/15/22)	389K	Pfizer First Quarter 2022 Earnings Teleconference[13]
Estimated total number of infections among high-risk individuals (7/09/22 – 7/15/22)	7.6 million	Calculated from Institute for Health Metrics and Evaluation COVID-19 Estimated Infections Data[11]
Proportion of Paxlovid uptake in general population (7/09/22 – 7/15/22)	5.1%	Calculated from the above 2 rows and 'Percent of patients who are high risk and qualify for Paxlovid'
Proportion of Paxlovid uptake in nursing home residents (September)	14.8%	Calculated from CMS Nursing Home Data[14]
Total Omicron hospitalizations (12/15-3/15)	1.1 million	Calculated from Our World in Data[15]
Total Omicron deaths (1/7-4/7)	150k	Calculated from Institute for Health Metrics and Evaluation COVID-19 Estimated Infections Data[11]

Model Parameter Explanations:

Because there remains substantial uncertainty in many parameter estimates, we vary estimates substantially, drawing on different sources and populations, in sensitivity analyses.

Probability patient is tested within 5 days of symptoms: The COVID-19 Case Surveillance Public Use Data from the CDC was used to calculate the proportion of patients with symptoms who received a COVID-19 test within 5 days of symptom onset. This probability was varied between 55% and 85% in Models 2-3 and 9-10 due to statewide variability in testing.

Probability of COVID-Like symptoms in US population: The Carnegie Mellon University Delphi Group COVID-Like Symptoms tracker was used to find the average proportion of the population with COVID-like symptoms between December 15, 2021 and March 15, 2022. This proportion was raised to 5% in Models 6 and 10 given higher proportions of COVID-Like Symptoms in certain states and among unvaccinated populations.

Percent of patients who are high risk and qualify for Paxlovid: This baseline figure was taken explicitly from Koma et al. Kaiser Family Foundation as the percent of adults who are at high risk of serious illness if infected with COVID-19. This value ranges from 30% (Model 9) in Utah to 49.3% (Model 10) in West Virginia.

US population: This is the current US population as presented by the US population clock rounded to the nearest million.

© 2023 Khunte M et al. *JAMA Health Forum*.

Duration of symptom episode in days: According to Menni et al. The Lancet, the average number of days of symptoms is 7. As such, 7 days was applied as the baseline. 8 days was used as an upper limit (Model 10), and 6 days was used as the lower limit (Model 9).

Average of number of tests for symptomatic individuals (infected): This value was computed as the inverse of the overall sensitivity of the rapid antigen test (65.3%), which means that on average 1.5 tests must be performed for a positive result in an infected individual.

Average of number of tests for symptomatic individuals (non-infected): The overall sensitivity of the rapid antigen test is 65.3% which means that a patient may have to take the COVID test more than once to rule out infection. The CDC specifies that a follow-up test may be required.

Average of number of tests for symptomatic individuals (total): This value was computed as the weighted average of the number of symptomatic tests for infected and uninfected individuals given the probability of a symptomatic patient being infected. This value ranges from 1.5, in the case of infected symptomatic individuals, to 2.4 in the case that uninfected symptomatic individuals use an average of 2.5 tests.

Reductions in overall hospitalization and mortality due to Paxlovid: The Arbel et al. study was used to derive the reductions in overall hospitalization and mortality. The study included more than 100,000 patients and had both vaccinated and unvaccinated patients.

Reductions in overall hospitalization and mortality due to Paxlovid in vaccinated population: Pfizer reported interim results in the format of a press release for their trial studying the effectiveness of Paxlovid in high-risk vaccinated individuals and standard risk unvaccinated adults (i.e., low risk of hospitalization or death).

Reductions in overall hospitalization and mortality due to Paxlovid in unvaccinated population: The Hammond et al study, the original study used to show the effectiveness of Paxlovid, was conducted in high-risk unvaccinated patients. The values from this publication are used for this parameter.

Estimated number of daily infections during Omicron Wave: The Institute for Health Metrics and Evaluation COVID-19 Estimated Infections Data was used to calculate the number of daily infections during the Omicron wave (12/15-3/15) by taking the average of all daily infections during this period.

Estimated number of total infections during Omicron Wave: The Institute for Health Metrics and Evaluation COVID-19 Estimated Infections Data was used to calculate the total number of infections during the Omicron wave (12/15-3/15) by summing all daily infections during this period.

Proportion of positive patients that are symptomatic: This proportion was determined by subtracting the proportion of asymptomatic infections (given in Ma et al. JAMA Infectious Diseases) from 1.

Proportion of symptomatic patients that are positive for COVID: This proportion was calculated using Bayes' Theorem given the proportion of positive patients that are symptomatic, the probability of COVID-like symptoms, and the probability of COVID infection (given by the ratio of daily infections during the Omicron wave and the US population).

Total courses of Paxlovid utilized (7/09/22 – 7/15/22): The total number of Paxlovid courses utilized from April 22 to April 28 was reported in Pfizer's First Quarter 2022 Earnings Teleconference.

© 2023 Khunte M et al. *JAMA Health Forum*.

Estimated total number of infections among high-risk individuals (7/09/22 – 7/15/22): The Institute for Health Metrics and Evaluation COVID-19 Estimated Infections Data was used to calculate the total number of infections during this period by summing all daily infections from 7/09/22 – 7/15/22. The number of infections were multiplied by the percentage of high-risk patients in the population.

Proportion of Paxlovid uptake in population (7/09/22 – 7/15/22): The total courses of Paxlovid utilized during 7/09/22 – 7/15/22 period was divided by the number of infections during this time frame.

Proportion of Paxlovid uptake in nursing home residents (September 2022): The Nursing Home Data from CMS was used to calculate the total number of new nursing home COVID-19 infections during September and total courses of Paxlovid administered in nursing homes during the same time period.

Total Omicron hospitalizations (12/15-3/15): This was calculated by summing the total new US hospital admissions between 12/15 and 3/15 using a dataset from Our World in Data.

Total Omicron deaths (1/7-4/7): This was calculated by subtracting the cumulative Omicron deaths in 1/7 from the cumulative Omicron deaths in 4/7, which are both provided by the Institute for Health Metrics and Evaluation COVID-19 Estimated Infections Data.

eTable 2: Model Number and Logic for Model

Model Number	Logic for Model
1	Base case scenario (expected values for each variable)
2	Lower end of estimates of Paxlovid effectiveness
3	Higher end of estimates of Paxlovid effectiveness
4	Estimates for Paxlovid effectiveness assuming the level of Paxlovid uptake found in nursing homes (12.8%)
5	Estimates for Paxlovid effectiveness assuming a vaccinated patient population
6	Estimates for Paxlovid effectiveness assuming an unvaccinated patient population
7	Estimates for Paxlovid effectiveness given an increase in Paxlovid uptake to 40%
8	Estimates for Paxlovid effectiveness given an increase in Paxlovid uptake to 80%
9	Lower end of estimates of number of symptomatic tests needed in Omicron-like wave
10	High end of estimates of number of symptomatic tests needed in Omicron-like wave

© 2023 Khunte M et al. *JAMA Health Forum*.

eTable 3: Formulae for calculated parameters in eTable 1

Parameter	Base Case Value	Formula
Probability patient is tested within 5 days of symptoms*	78%	$\frac{\text{Sum of symptomatic patients tested within 5 days}}{\text{Sum of symptomatic patients}}$
Average number of tests for Symptomatic Individuals (infected)	1.5	$\frac{1}{\text{Rapid Antigen Test Sensitivity}}$
Average number of tests for symptomatic Individuals (total)	1.95	$\left(\frac{\text{Proportion of positive symptomatic patients}}{\text{Rapid antigen test sensitivity}}\right) + (\text{Proportion of negative symptomatic patients}) \times (\text{Average number of tests for negative symptomatic patients})$
Estimated number of daily infections during Omicron Wave*	1.9M	Mean of daily US Infections from 12/15/2021 to 3/15/2022
Estimated number of total infections during Omicron Wave*	169M	Sum of daily US Infections from 12/15/2021 to 3/15/2022
Proportion of positive patients that are symptomatic	59.5%	$1 - \text{percentage of asymptomatic infections}$
Proportion of symptomatic patients that are positive for COVID	11%	$\frac{\text{Proportion of symptomatic infections} \times \text{Daily US infections}}{\text{Probability of CLI} \times \text{US population}}$
Estimated total number of infections (7/09/22 – 7/15/22)*	20.2M	Sum of daily US Infections from $7/09/22 - 7/15/22$
Proportion of Paxlovid uptake in general population (7/09/22 – 7/15/22)	5.1%	$\frac{\text{Total Paxlovid utilization in July}}{\text{Total infections in July} \times \text{Percent of Paxlovid-eligible patients}}$
Proportion of Paxlovid uptake in nursing homes (NH) (September)*	14.8%	$\frac{\text{Sum of US Paxlovid NH uptake in September}}{\text{Sum of US New NH infections in September}}$
Total Omicron hospitalizations (12/15-3/15)*	1.1M	Sum of US hospital admissions from 12/15/2021 to 3/15/2022
Total Omicron deaths (1/7-4/7)*	150k	Cumulative deaths in 1/7 $-$ Cumulative deaths in 4/7

*Code available on Github at: https://github.com/mkhunte/test_to_treat

© 2023 Khunte M et al. *JAMA Health Forum*.

eReferences

1. *COVID-19 Case Surveillance Public Use Data*, in *Centers for Disease Control and Prevention*. 2022: https://data.cdc.gov/Case-Surveillance/COVID-19-Case-Surveillance-Public-Use-Data/vbim-akqf.
2. *COVID-Like Symptoms*, in *Carnegie Mellon University Delphi Group* 2022: https://delphi.cmu.edu/covidcast/indicator/?sensor=fb-survey-smoothed_wcli.
3. Koma, W., et al., *How Many Adults Are at Risk of Serious Illness If Infected with Coronavirus? Updated Data.* Kaiser Family Foundation, 2020.
4. *U.S. and World Population Clock*. 2022, United States Census Bureau: https://www.census.gov/popclock/.
5. Menni, C., et al., *Symptom prevalence, duration, and risk of hospital admission in individuals infected with SARS-CoV-2 during periods of omicron and delta variant dominance: a prospective observational study from the ZOE COVID Study.* Lancet, 2022. **399**(10335): p. 1618-1624.
6. Jegerlehner, S., et al., *Diagnostic accuracy of a SARS-CoV-2 rapid antigen test in real-life clinical settings.* Int J Infect Dis, 2021. **109**: p. 118-122.
7. *COVID-19 Testing: What You Need to Know*. Centers for Disease Control and Prevention 2022; Available from: https://www.cdc.gov/coronavirus/2019-ncov/symptoms-testing/testing.html.
8. Arbel, R., et al., *Oral Nirmatrelvir and Severe Covid-19 Outcomes During the Omicron Surge.* Research Square, 2022.
9. *Pfizer Announces Additional Phase 2/3 Study Results Confirming Robust Efficacy of Novel COVID-19 Oral Antiviral Treatment Candidate in Reducing Risk of Hospitalization or Death*. 2021, Pfizer: https://www.pfizer.com/news/press-release/press-release-detail/pfizer-announces-additional-phase-23-study-results.
10. Hammond, J., et al., *Oral Nirmatrelvir for High-Risk, Nonhospitalized Adults with Covid-19.* N Engl J Med, 2022. **386**(15): p. 1397-1408.
11. *COVID-19 Projections*. 2022, Institute for Health Metrics and Evaluation: https://covid19.healthdata.org/united-states-of-america.
12. Ma, Q., et al., *Global Percentage of Asymptomatic SARS-CoV-2 Infections Among the Tested Population and Individuals With Confirmed COVID-19 Diagnosis: A Systematic Review and Meta-analysis.* JAMA Netw Open, 2021. **4**(12): p. e2137257.
13. *Pfizer First Quarter 2022 Earnings Teleconference*. 2022: https://investors.pfizer.com/.
14. *COVID-19 Nursing Home Data*. 2022, Centers for Medicare & Medicaid Services: https://data.cms.gov/covid-19/covid-19-nursing-home-data.
15. *Coronavirus (COVID-19) Hospitalizations*. 2022, Our World in Data: https://ourworldindata.org/covid-hospitalizations#covid-19-hospitalizations.

© 2023 Khunte M et al. *JAMA Health Forum*.
