# Supplementary material for: Projected COVID-19 Mortality Reduction From Paxlovid Rollout
Source: JAMA Health Forum. 2023 Mar 17;4(3):e230046. doi: 10.1001/jamahealthforum.2023.0046 (PMC10024200; doi:10.1001/jamahealthforum.2023.0046)
Supplement: Supplement 2. — Data Sharing Statement [file jamahealthforum-e230046-s002.pdf]

# Data Sharing Statement

Khunte. Projected COVID-19 Mortality Reduction From Paxlovid Rollout. *JAMA Health Forum*. Published March 17, 2023. doi:10.1001/jamahealthforum.2023.0046

## Data
**Data available:** Yes
**Data types:** Data (not involving human participants)
**How to access data:** The data sources are listed in the Supplement. The corresponding author can be reached at alyssa_bilinski@brown.edu.
**When available:** With publication

## Supporting Documents
**Document types:** Statistical/analytic code
**How to access documents:** alyssa_bilinski@brown.edu
**When available:** With publication

## Additional Information
**Who can access the data:** anyone requesting the data
**Types of analyses:** for any purpose
**Mechanisms of data availability:** with investigator support
